# Newly Established Monoclonal Antibody Diagnostic Assays for *Schistosoma mansoni* Direct Detection in Areas of Low Endemicity

**DOI:** 10.1371/journal.pone.0087777

**Published:** 2014-01-31

**Authors:** Rafaella Fortini Queiroz Grenfell, Paulo Marcos Zech Coelho, Diana Taboada, Ana Carolina Alves de Mattos, Ruth Davis, Donald A. Harn

**Affiliations:** 1 Schistosomiasis Laboratory, Rene Rachou Research Center, Oswaldo Cruz Foundation (Fiocruz), Belo Horizonte, Minas Gerais, Brazil; 2 Department of Infectious Diseases, College of Veterinary Medicine, and Center for Tropical and Emerging Global Diseases, University of Georgia (UGA), Athens, Georgia, United States of America; 3 Monoclonal Antibody Facility, College of Veterinary Medicine, University of Georgia (UGA), Athens, Georgia, United States of America; New York University, United States of America

## Abstract

**Background:**

Current available methods for diagnosis of schistosomiasis mansoni lack sufficient sensitivity, which results in underreporting of infectious in areas of low endemicity.

**Methodology/Principal Findings:**

We developed three novel diagnostic methodologies for the direct detection of schistosome infection in serum samples. These three new methods were evaluated with positive patients from a low endemicity area in southeast Brazil. The basis of the assay was the production of monoclonal antibodies against the protein backbone of heavily glycosylated Circulating Cathodic Antigen (CCA). The antibodies were also selected for having no specificity to repeating poly-*Lewis x* units. Assays based on the detection CCA-protein should not encounter a limitation in sensitivity due to a biological background of this particular epitope. Three diagnostic methodologies were developed and validated, (i) Immunomagnetic Separation based on improved incubation steps of non-diluted serum, (ii) Direct Enzyme-linked Immunosorbent Assay and (iii) Fluorescent Microscopy Analysis as a qualitative assay. The two quantitative assays presented high sensitivity (94% and 92%, respectively) and specificity (100%), equivalent to the analysis of 3 stool samples and 16 slides by Kato-Katz, showing promising results on the determination of cure.

**Conclusions/Significance:**

The Immunomagnetic Separation technique showed excellent correlation with parasite burden by Cohen coefficient. The qualitative method detected 47 positive individuals out of 50 with the analysis of 3 slides. This easy-to-do method was capable of discriminating positive from negative cases, even for patients with low parasite burden.

## Introduction

Schistosomiasis mansoni is a major parasitic disease associated with considerable morbidity and mortality in the developing world and may lead to sequelae of acute and chronic infection [1]. The gold standard for diagnosis of schistosomiasis mansoni is the detection of characteristic parasite eggs in stools. The direct detection of eggs is difficult and not always possible in patients with low parasite burdens, with consequent attainment of low egg-shedding rates. Serological tests are widely used to detect antibodies against worm or soluble egg antigens. However, these assays are unable to differentiate between persistent and inactive infection [2]. While polymerase chain reaction methods can detect schistosomal DNA none of the published PCR methods has been evaluated for utilization in routine diagnosis [3], [4].

Measurement of circulating antigens, as Circulating Cathodic Antigen (CCA) that are genus-specific glycoconjugates associated with the gut of the worm, appears promising [5]–[8]. CCA is regurgitated from worms into the circulatory system and levels of CCA are related to the presence and intensity of schistosome infection [9], [10]. However, the techniques for measuring circulating antigens proved to be cumbersome and had a low level of sensitivity [8], [9]. The most recent advancement of the CCA test is a CCA dipstick for use with urine samples [11]. In tests for *S. mansoni* in Africa, the urine-CCA cassette test has shown to be more sensitive than single or triple Kato-Katz method on a single faecal specimen in moderate and high prevalence areas [12]–[16]. Multivariable modeling estimated POC-CCA as significantly more sensitive than Kato-Katz at low infection intensities (<100 eggs/gram stool) when one sample was tested [16]. Although low prevalence areas are still a challenge, the CCA cassette test can be recommended for the rapid identification of *S. mansoni* infections before treatment. Contrarily, it is important to consider that assays based on the detection of CCA glycans should encounter a limitation in sensitivity due to a biological background of the glycoconjugates. Native CCA glycoprotein contains 0-linked poly (*Lewis X*) carbohydrate chains with approximately 25 repeating units. Carbohydrate chains containing multiple *Lewis X* determinants have been identified on several glycolipids not only from schistosomes but also from other parasites, from human adenocarcinomas and also, circulating granulocytes carry relatively high abundance of branched N-linked polysaccharides having *Lewis X* repeating units, as previously discussed [17]. Another alternative is the detection of *Schistosoma* sp. Circular Anodic Antigen (CAA) considered a valuable tool for population screening and epidemiological research and, in contrast to the CCA carbohydrate, CAA carbohydrate does not contain *Lewis X* repeating units. A lateral flow assay developed to test CAA in serum for individual diagnosis of imported active schistosome infections demonstrated to be a low-complexity test with higher sensitivity than the CAA-ELISA [18].

Fluorescence imaging has also become a valuable approach for antigen detection [19], [20]. For utilization under field conditions, an assay should be rapid, specific and, most importantly, sensitive enough to discriminate between active infections [21], [22].

Our group has recently developed new methodologies for the diagnosis of schistosomiasis mansoni based on the detection of antibodies in serum samples using CCA “crude antigen”, recombinant CCA and CCA peptides [17]. The current study evaluates the potential of three new diagnostic methods for schistosome infection, each based on the direct detection of CCA for determining active schistosome infection and to monitor the effectiveness of therapy. The new methods take advantage of (i) paramagnetic microspheres that are then coated with the anti-CCA monoclonal antibody for the specific detection of CCA epitopes, that are not of the *Lewis x* epitope, reducing potential cross-reactivity with carbohydrates; (ii) incubation steps under rotation enhancing the binding of antigen-antibody; (iii) use of non-diluted serum permitting the detection even in patients with low parasite burdens. To test these concepts, individuals from an area of low endemicity were selected and intensively monitored by parasitological assays before and after therapy prior to the analysis of the new methods.

## Materials and Methods

### Ethics Statement

This project was approved by the Ethical Research Committee of Fiocruz for animal use (CEUA-L0023/08) according to the International Guiding Principles for Biomedical Research Involving Animals developed by the Council for International Organizations of Medical Sciences. The Ethical Research Committee of Fiocruz (CEPSH-03/2008) and the National Brazilian Ethical Board (784/2008, CONEP-14886) approved the human study. The study objectives were explained to all participants and written consent was obtained in writing before admission to this study. Parents/guardians provided written consent on behalf of child participants.

### Community Survey

We performed a study in Buriti Seco and Morro Grande in Pedra Preta, a schistosomiasis endemic area in the rural region of Montes Claros, southeast Brazil [23]. This population has a low population migration index and has never been treated for schistosomiasis. The number of residents participating in the survey was 201 individuals aged 1 to 88 (93 female and 108 male).

Four stool samples per individual were collected on four consecutive days for Kato-Katz analysis [24]. This method was performed using 18 slides, which were prepared as follows for each participant: 12 slides of the first sample and two slides each for the second, third and fourth sample in a total of 750 mg of faeces (18×42.7 mg). Samples were also analyzed by quantitative TF-Test [25]. Briefly, samples were passed through a nylon mesh and quantified in metal plates (500 mg). Each 500 mg portion was transferred to a tube with preservative solution (10% formalin) (Vetec; Rio de Janeiro, Brazil) and processed using neat ethyl acetate. Samples were centrifuged at 500 g for 2 min. The sediment was resuspended in 0.85% saline solution and analyzed using optical microscopy. TF-Test was repeated three times for each sample, with a total of 1500 mg of stool sample and results were equivalent to 36 Kato-Katz slides.

Among the individuals participating in the survey, fifty patients aged 8–88 were selected to provide serum samples (24 female and 26 male). Among these individuals, egg output ranged from 1 to 555 eggs per gram of faeces (EPG). Blood was collected by venous puncture and individual serum samples obtained after centrifugation of blood samples at 3000 g for 5 min. Samples were maintained at –20°C.

All participants with egg-patent schistosomiasis were treated with single dose of praziquantel; 60 mg/kg for children and 50 mg/kg for adults, as recommended by the Brazilian Ministry of Health. Infections with other helminthes were treated with 400 mg albendazole. Thirty six positive patients were re-examined by Kato-Katz (18 slides) and TF-Test 30 and 90 days post therapy and retreated as needed; serum samples were also obtained for these individuals. A schematic showing the distribution of subjects and detection of infection is shown in S1.

### Healthy Donors

Fifty two schistosomiasis-negative volunteers aged 22 to 65 (34 female and 18 male) were selected to be part of the negative control group. These volunteers were residents in non-endemic areas with no history of previous schistosome infection. Serum samples of these donors were processed as described earlier.

Besides the historical criteria used to select healthy donors, we also performed ELISA assays for the detection of IgG antibodies against soluble adult worm antigens (ELISA-SWAP) and soluble egg antigens (ELISA-SEA).

### Production of Monoclonal Antibody Specific for CCA-protein (mAbCCA)

Nine-week-old female BALB/c mice were subcutaneously immunized with 0.1 mg of the CCA “crude antigen” previously obtained [17] using a new vaccine delivery method (US patent n.61/476,431) as adjuvant. Two weeks later, mice were boosted. Serum from mice was tested by ELISA to determine the antibody titer against the antigen. Mice were given an additional boost 15 days after the first boost by intraperitoneal injection of 0.1 mg of CCA “crude antigen”. Three days later, spleen cells were fused with Sp2/O-Ag14 myeloma cells using polyethyleneglycol. The fused cells were cultured at 37°C and selected with hypoxanthine-aminopterin-thymidine medium (Sigma-Aldrich; St. Louis, United States of America) (HAT). The initial screen of positive growth wells was made by ELISA. Antigen was diluted 1 µg/ml in 0.05 M carbonate-bicarbonate buffer pH 9.6 (coating buffer) and microtiter plates MaxiSorp Surface (NUNC, Thermo Scientific; Roskilde, Denmark) were coated at 4°C for 16 h. After blocking, 100 µl of culture supernatants of the HAT-selected hybridomas were added and incubated for 1 h at room temperature. The bound antibodies were detected using peroxidase-conjugated anti-mouse IgG (1∶5,000) after incubation for 1 h at room temperature (Southern Biotech; New Orleans, United States of America) and 100 µl of substrate 3,3′,5,5-tetramethylbenzidine solution (TMB) (Invitrogen; Grand Island, United States of America). ELISA positive hybridomas were selected. A second ELISA was performed to differentiate the hybridomas producing mAbs against the *Lewis x* epitope. Plates were coated with *Lewis x* tetrasacharide (Sigma-Aldrich; Saint Louis, United States of America) and the ELISA performed as described. Hybridomas non-reactive for *Lewis x* were selected and Ig-subclasses were determined by a kit for monoclonal isotyping (Sigma-Aldrich; Saint Louis, United States of America) (Table 1).

**Table 1:** Characterization of mAbs produced against antigens of purified CCA glycoprotein.

Clones	Ig-subclass	ELISA	
		CCA specificity	*Lewis x* specificity
1.3C2b	IgG1	+++	–
1.2C6	IgG1	++	–
4.4C3	IgG1	+++	–
5.1B4	IgG1	+++	–
5.1B1	ND	++	+++
5.2A3	IgG1	++	–
5.1D3	IgG1	++	–
5F4.E4	ND	++	++
16D7.C10	IgM	+++	–
16D7.C4	ND	+++	++
16D7.B9	ND	+++	++
12D3.F2	IgM	+++	–
12D3.G8	ND	++	++

−: negative reaction (OD<0,033); ++: positive reaction (OD<0,330); +++: positive reaction (OD<0,660); ND = not determined.

The selected clones (16D7.C10 IgM and 5.1B4 IgG1) was grown in hybridoma medium (Invitrogen; Grand Island, United States of America) supplemented with penicillin (100 U/ml) and streptomycin (100 mg/ml) (Invitrogen; Grand Island, United States of America) at 37°C. Culture supernatants were harvested in a final volume of 1 liter by inverting flask into collection tube without disturbing the cells and used for ammonium sulfate precipitation [26]. Precipitated proteins were dialyzed against PBS and then mAbs were purified by protein G purification column (Sigma-Aldrich; Saint Louis, United States of America). After measuring the OD at 280 nm of the fractions, the protein-containing fractions were stored at −20°C. Aliquots of the mAbs (16D7.C10) were conjugated to peroxidase with Zenon Mouse Labeling Kit (Invitrogen; Grand Island, United States of America), and also to Alexa Fluor 647 with Fluorochrome Protein Labeling Kit (Invitrogen; Grand Island, United States of America).

### Immunological Assay Evaluation for the Direct Detection of CCA in Individual Serum

#### Immunomagnetic separation technique with mAbCCA-protein (IMS-mAbCCA)

IMS was standardized according to the previously described [17] with some modifications. Paramagnetic microspheres (0.4 µm; 10^6^ microspheres/assay) (Estapor Microspheres, Merck; Lyon, France) were sensitized with 1 µg/ml of mAbCCA-protein (5.1B4) in coating buffer for a physical adsorption. The following steps were performed with a rotating suspension system to improve antigen-antibody binding. Microspheres were incubated for 16 h at 4°C, and then washed four times with 500 µl of 0.15 M phosphate buffer saline with 0.05% tween pH 7.2 (washing buffer) using a 1.5 ml tube magnetic base (Invitrogen; Grand Island, United States of America). During washing steps, microspheres stays in contact with the magnetic base for 10 sec when washing buffer are removed. Nonspecific sites were blocked with 20% skim milk at 4°C for 16 h. The microspheres were then maintained at 4°C until use. On the day of the analysis the microspheres were washed, and then 200 µl of undiluted serum samples were added into duplicate tubes, followed by incubation at 37°C for 2 h. Microspheres were washed, then incubated at 37°C for an hour with 100 µl of peroxidase conjugated mAbCCA-protein (16D7.C10) diluted 1∶400. Each tube was washed again and 100 µl of TMB (Invitrogen; Grand Island, United States of America) were added to each well. The reaction was stopped after 10 min incubation by addition of 100 µl/tube of 2 N sulfuric acid. A magnetic base was used to separate the beads from the supernatant, which was transferred to a microtiter plate and results were obtained at 450 nm in a microplate reader (Model 3550, Bio-Rad Laboratories; Tokyo, Japan).

#### ELISA with mAbCCA-protein (ELISA-mAbCCA)

ELISA was standardized based on the technique previously described [17] with some modifications. Microtiter plates MaxiSorp Surface (NUNC, Thermo Scientific; Roskilde, Denmark) were coated with mAbCCA-protein (5.1B4) 1 µg/ml in coating buffer. Plates were washed and blocked by addition of 300 µl per well of 2.5% skim milk, incubating at 37°C for 1 h. After additional washing, 100 µl of individual serum diluted 1∶100 was added to the plate wells in duplicate and incubated for 1 h. Plates were then incubated with peroxidase conjugated mAbCCA-protein diluted 1∶400 followed by addition of TMB (Invitrogen; Grand Island, United States of America). The enzymatic reaction stopped after 10 min of incubation in the dark with 50 µl/well of 2 N sulfuric acid and the absorbance measured at 450 nm in microplate reader (Model 3550, Bio-Rad Laboratories; Tokyo, Japan).

#### Fluorescent microscopy analysis of IMS products using mAbCCA-protein (FluoIMS-mAbCCA)

The same procedure adopted for IMS*-*mAbCCA was used here for a double blind analysis. After microspheres were sensitized and blocked, 200 µl of serum samples were added and incubated for 1 h at 37°C. Microspheres were then washed and 100 µl of Alexa Fluor 647 conjugated mAbCCA-protein (16D7.C10) (1∶400) were added followed by an incubation for 1 h at 37°C. Qualitative analysis of 5 µl of microspheres suspension was performed by examination on a glass slide with a fluorescent microscope (Karl Zeiss; Oberkochen, Germany) to visualize fluorescent microspheres (642 nm, emission filter LP590). Photographic records were taken with a digital camera (Canon EOS).

Positive and negative controls were assayed for each technique as control of nonspecific adsorption of conjugate.

### Statistical Analysis

Absorbance value data were analyzed with Minitab Inc. by Kolmogorov-Smirnov normality test. Normal distributed data were analyzed by Student’s *t* test and non-normal data by Mann-Whitney test. Comparisons between methods were done by 2 proportions’ Fisher analysis (p≤0.05 as significance level). Sensitivity, specificity and cut-off values were determined with Prism 4.0. Agreement between methods was measured using Cohen coefficient [27] and analyzed by Landis & Koch definition [28].

## Results

### Generation of CCA-protein-specific mAbs

CCA “crude antigen” previously obtained from *S. mansoni* adult worm extract [17] was used for mice immunization. The fusion of splenocytes from these mice with Sp2/0-Ag14 myeloma cells yielded a total of 186 HAT-resistant hybridoma clones. Thirteen clones with high CCA-protein binding as determined by ELISA were selected. Among these thirteen, 5 clones were eliminated as they bound to *Lewis x*. Clones secreting mAbs against other CCA-protein epitopes were then isotyped, expanded and stored in liquid nitrogen. The characterization of the mAbs reacting specifically with CCA-protein antigens is summarized in Table 1.

### Validation of IMS-mAbCCA and ELISA-mAbCCA as Quantitative Methods for the Diagnosis of Schistosomiasis Mansoni

Fifty patients from Pedra Preta with an egg-output of 1 to 555 EPG were selected to provide serum samples, together with fifty two negative individuals (non-endemic area residents). Table 2 presents the parasitological diagnosis of the 50 endemic individuals determined by Kato-Katz reporting the prevalence after the first, second, third and fourth stool samples.

**Table 2:** The number of stool samples analyzed by Kato-Katz thick smears required for the positive diagnosis of endemic individuals.

	First sample				Second sample	Third sample	Fourth sample
Kato-Katz Number of slides	1	2	7	12	14	16	18
Number of positiveindividuals (n = 50)	17	18	24	30	30	40	50

**Figure 1:**
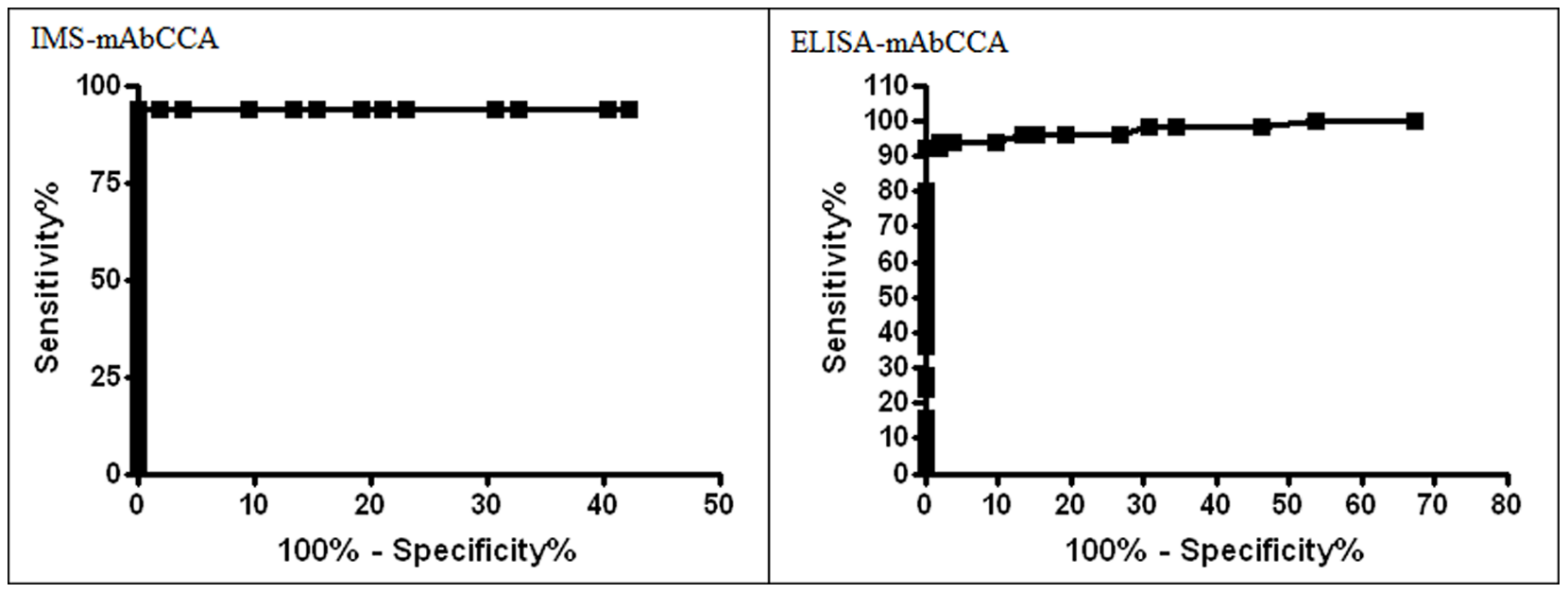
ROC curves for the quantitative methodologies based on the direct detection of CCA in serum. IMS-mAbCCA (A = 0.957, p<0.0001) and ELISA-mAbCCA (A = 0.982, p<0.0001). Artwork created by Prism 5.0 software.

**Figure 2:**
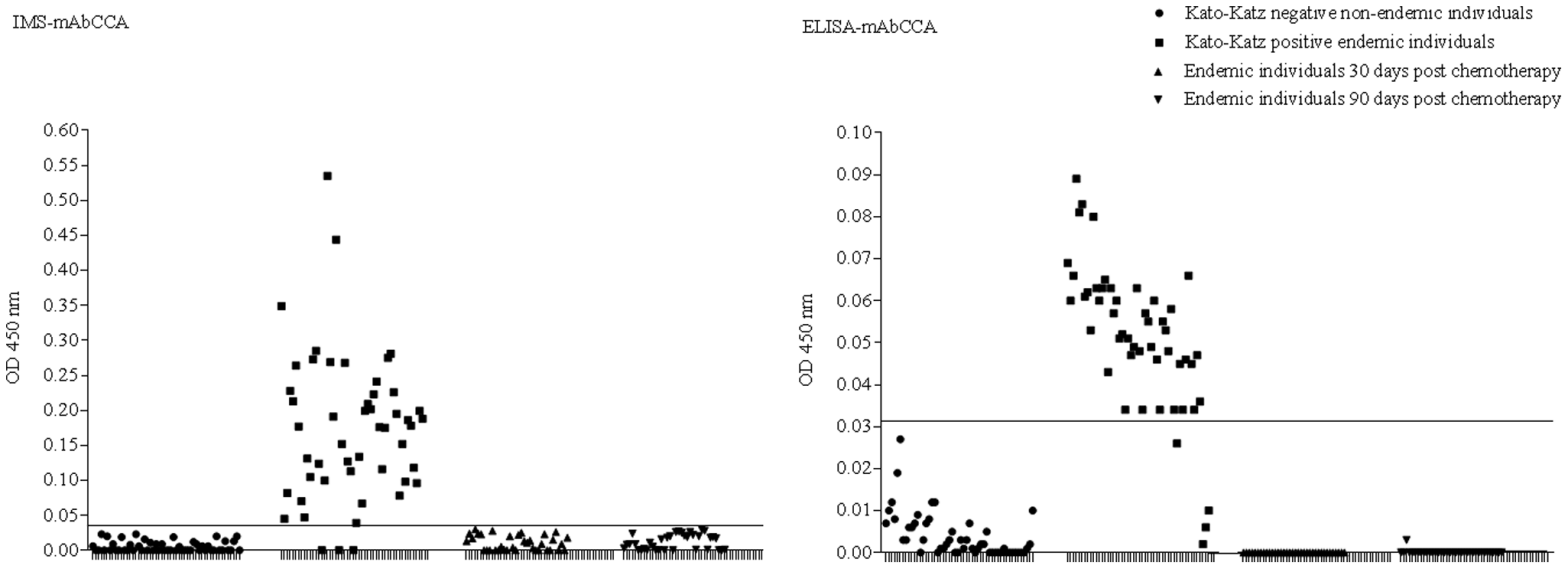
Individual analysis of serum samples by IMS-mAbCCA and ELISA-mAbCCA protocols. Each OD value is representative for the mean of four absorbance values. Groups are represented by 50 positive individuals from Pedra Preta, 52 negative individuals and 36 patients after 30 and 90-off values are represented by bars and were defined by the ROC curves. IMS-mAbCCA (cut-off = 0.036) and ELISA (cut-off = 0.031). Artwork created by Prism 5.0 software.

Initial steps allowed the determination of the cut-off values, positivity ratios, sensitivity and specificity of both methodologies by using a ROC curve analysis, represented in Figure 1. The IMS methodology demonstrated 94% sensitivity and 100% specificity (cut-off = 0.036), whereas ELISA showed similar effectiveness with 92% and 100% sensitivity and specificity, respectively (cut-off = 0.031). No false-positives were seen, but IMS-mAbCCA presented three false-negative while ELISA-mAbCCA presented four false-negative results. Analysis of the positivity ratios were 97% (99/102) for IMS and 96% (98/102) for ELISA. Thirty six of the selected positive patients from Pedra Preta donated serum and fecal samples 30 and 90 days after therapy. Fecal samples were intensively revaluated by Kato-Katz and TF-Test as described and no eggs were found in any sample 30 days after therapy. On the other hand, two patients presented one EPG after 90 days of therapy. Neither of these two patients was detected as positive by either the IMS or ELISA assays (Figure 2, Figure S1). Individual OD results determined by each quantitative methodology for each patient are shown in Figure 2.

Analysis of Cohen’s Kappa Index showed an almost perfect agreement of 0.941 (±0.099) between parasitological results and IMS-mAbCCA. A similar result was found for ELISA-mAbCCA in comparison to Kato-Katz with an agreement of 0.921 (±0.099). The same agreement was found when IMS-mAbCCA was compared to ELISA-mAbCCA (kappa index = 0.921±0.099). A positive correlation between IMS-mAbCCA results and fecal egg output for the fifty positive patients was found (R^2^ = 0.99). Three exceptions for the positive correlation were found for patients that presented 4, 7 and 39 EPG that were not detected by IMS-mAbCCA. ELISA-mAbCCA did not show the same positive correlation between absorbance values and egg counts (data not shown) than IMS.

To better compare the sensitivity of the new methodologies with Kato-Katz, a comparison was done with different numbers of Kato-Katz slides from each endemic patient. Kato-Katz analysis revealed that after 2 slides of the same fecal sample, only 18 of the 50 selected patients presented eggs in stool. After 7 and 12 Kato-Katz slides, the number of positive patients increased to 24 and 30, respectively. Only after the analysis of fecal samples collected for four consecutive days, all the 50 patients were positive by Kato-Katz.

### Validation of FluoIMS-mAbCCA as a Qualitative Method for the Diagnosis of Schistosomiasis Mansoni

**Figure 3:**
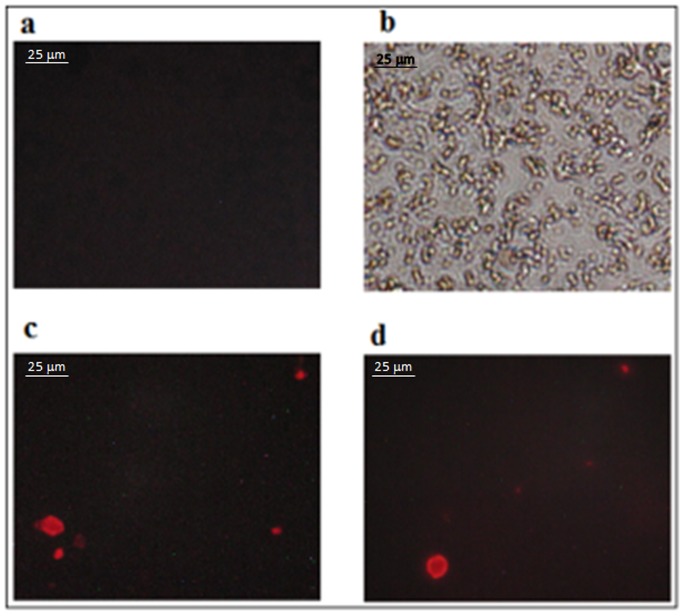
Representative images of FluoIMS-mAbCCA. In (a) negative serum sample, (b) microspheres visualization under white light, (c) and (d) positive serum samples with fluorescent microspheres under 642 nm, emission filter LP 590.

Serum samples from positive and negative individuals were also used for the standardization of a qualitative method. By using Alexa fluor 647 attached to a second specific mAb, microspheres could be visualized using fluorescent microscopy. Analysis showed that the microspheres with small size of 0.4 µm decreased the visibility creating a limitation for this assay. Identification of positive microspheres can be seen in Figure 3.

Nonetheless, the positivity ratio for FluoIMS was 74% (75/102) when one slide was performed. Thirty two positive individuals out of fifty and forty three negative individuals out of fifty two were properly detected. Moreover, six false-positive results were seen for the thirty six samples obtained after 30 days of therapy, decreasing the specificity of the assay. The accuracy of FluoIMS was increased when two extra slides were performed for each false-negative result for the three patients mistakenly diagnosed (*p*<0.001). Completed data are shown in Table 3.

**Table 3:** Performance of FluoIMS-mAbCCA on the detection of CCA in patients’ sera.

Individuals	Results with 1 slide				Results with 3 slides						
	*n*		%		*n*			%			
	+	–	+	–	+	–		+		–	
Positive (*n* = 50)	32***	18	64	36	47***		3		94	6	
Post therapy (30 days) (*n* = 36)^2^	3	33	8	92	ND^1^		ND		ND	ND	
Negative (*n* = 52)	9	43	17	83	ND		ND		ND	ND	

Comparison analysis performed using Kato-Katz and TF-Test results as gold standards.

1 ND = not determined.

*** represents statistical differences between 1 and 3 slides of FluoIMS (*p*<0.001).

2 36 out of 50 patients agreed to donate serum and faeces samples after treatment.

## Discussion

Identification of populations to be targeted for individual treatment and broad-spectrum therapy in schistosomiasis-endemic areas, assessment of therapy efficacy, morbidity, and evaluation of control strategies need to be based on reliable and available diagnostic tools [29]. Fecal detection lacks sufficient sensitivity and patient compliance [30]–[32], especially in areas where the endemicity is low and poor sensitivity limits application in large-scale and individual diagnosis [29], [33].

We recently developed and standardized new methodologies for the diagnosis of schistosomiasis mansoni based on the detection of antibodies in serum samples using CCA “crude antigen”, recombinant CCA and CCA peptides [17]. Although the new methodologies demonstrated high sensitivity and specificity and this antigen worked as a good marker for control of cure after praziquantel treatment, indirect serologic tests cannot differentiate active from treated infections in surveillance and thus cannot identify reinfection. Detection of circulating antigens secreted by living parasites has been considered an alternative diagnostic method to distinguish active infections [34]–. Therefore, our group developed new methodologies in which the direct detection of *S. mansoni* antigen in serum is provided by an extra incubation of non-diluted sera samples under rotation and the use of mAbs that lead to enhanced sensitivity and specificity. We validated the quantitative IMS methodology and results were compared to ELISA. The use of similar microsphere immunoassays has become a popular approach for the diagnosis of many food-borne and infectious diseases [37], [38].

Previously obtained CCA “crude antigen” [17] allowed the production of specific monoclonal antibodies. A clone that did not bind to the *Lewis x* portion of the antigen was selected. Native CCA glycoprotein was described in terms of an O-linked poly or *Lewis x* carbohydrate chain with approximately 25 repeating units. Carbohydrate chains containing multiple *Lewis x* determinants have been identified on several glycolipids not only from schistosomes but from other parasites [39], [40], human adenocarcinomas [41], and also circulating granulocytes carry branched N-linked polysaccharides having *Lewis x* repeating units [42]. Thus, the use of mAbs against the glycan epitopes of schistosome CCA might lead to false-positives, especially when *Lewis x* from other sources is detected [5]–[8], [13], [43].

Since schistosomiasis epidemiological profile has shown an increase in the number of low endemicity areas, the reliability of each methodology was validated with patients’ from an endemic area in southeast Brazil, where a low parasite burden was confirmed after an intensive and extensive analysis of fecal examination. Patients in that area had never been treated for schistosomiasis and had not been evaluated in a survey based on reevaluations and treatment schemes every time a reinfection case is detected.

Both IMS-mAbCCA and ELISA-mAbCCA demonstrated high sensitivity and specificity (Figure 1). The use of the new methods utilizing the CCA-protein specific IgM mAb yielded three false-negative results for IMS-mAbCCA and four for ELISA-mAbCCA (4–39 EPG) (Figure 2). Comparison between both methods with results obtained by Kato-Katz of the 50 endemic individuals after the first, second, third and fourth stool samples analyses demonstrated that at least 16 slides of 3 stool samples are required to reach the same sensitivity as the IMS-mAbCCA and ELISA-mAbCCA (Table 2). Similar results were previously found when the comparison between different numbers of Kato-Katz slides was performed to determine the prevalence of an endemic area in Brazil. Prevalence of Schistosomiasis mansoni was 12.5% based on two slides, 27.2% based on 10 slides of three stool samples and 35.4% based on 12 slides of four stool samples [32].

Analysis of drug treated patients demonstrated that both methods were consistent with the observed parasitological results. These data corroborate previous findings where the level of circulating antigens in serum drops rapidly after three to six weeks of successful therapy [10], [44], showing that CCA can be used as a relevant marker for the determination of cure. Two patients presented one EPG after 90 days post therapy and were not detected by IMS-mAbCCA or ELISA-mAbCCA (Figure 2). It must be of consideration that it is possible that these two patients were not compliant with the therapy or were reinfected. Complementary data showed that IMS, but not ELISA, presented a positive correlation between the light absorbency intensity and egg output (R^2^ = 0.99). Others [45] showed that the amount of circulating schistosome antigen was closely related to worm burden in infected mice [46].

Even with the established similarity of IMS-mAbCCA and ELISA-mAbCCA reliability for detecting active schistosomiasis infection, the superiority of IMS was revealed by its simplicity when used in field trials examining the impact of mass drug administration. The total time required for IMS was 2.3 hours in comparison to 4 hours for ELISA examination.

To date, qualitative methods have not been demonstrated for the detection of circulating antigens, despite the advantages this approach has in reducing assay time. We developed and validated the FluoIMS based on the same procedure described for IMS-mAbCCA except for the addition of the second mAb labeled here with Alexa fluor 647 for fluorescence microscopy analysis (Figure 3). Final data of a double blind analysis showed that an enhanced sensitivity of 94% was seen after the evaluation of 3 slides of each positive patient (*p*<0.001). On the other hand, the analysis of a single slide was enough to achieve 92% specificity for negative cases and 83% sensitivity for patients evaluated after therapy (Table 3). In conclusion, the reliability and positivity ratio of FluoIMS was significantly increased by two extra slides performed for each false-negative result (*p*<0.001) when 15 positive patients become detectable. Although promising, FluoIMS could be improved by making the detection of the microspheres easier. Plus, false-positive results may be reduced by increasing washing steps after incubation to reduce the background. Finally, a quantification of the fluorescent microspheres could be further standardized.

The present study was undertaken to standardize new schistosome diagnostic assays with significant accuracy for the diagnosis of hard-to-detect individuals. A special concern was to validate these new methodologies for their capability of discriminate active infections from previous contact using samples from low endemicity areas. The difficulty of diagnosing patients with low parasite burden represents an pressing medical need, since low endemicity areas will increase in number as control programs continue to use a small number of parasitological slides. Our study presents three methods with high sensitivity and specificity for the direct diagnosis of patients with low parasite burden. Although these assays are easy to perform, they require a rotator, a fluorescent microscope and/or a spectrophotometer and are limited to reference labs for the diagnosis of new low intensity infections in travelers or individuals living in endemic areas.

## Supporting Information

Figure S1 **Flowchart.** Diagram that represents the sequencing of operations for the prospective study performed in the communities of Buriti Seco and Morro Grande in Pedra Preta, Brazil.
(PDF)

Table S1 **STARD Checklist.** STARD checklist for reporting the study of diagnostic accuracy.
(PDF)
