# Supplementary figures and images for: Newly Established Monoclonal Antibody Diagnostic Assays for Schistosoma mansoni Direct Detection in Areas of Low Endemicity
Source: PLoS One. 2014 Jan 31;9(1):e87777. doi: 10.1371/journal.pone.0087777 (PMC3909226; doi:10.1371/journal.pone.0087777)

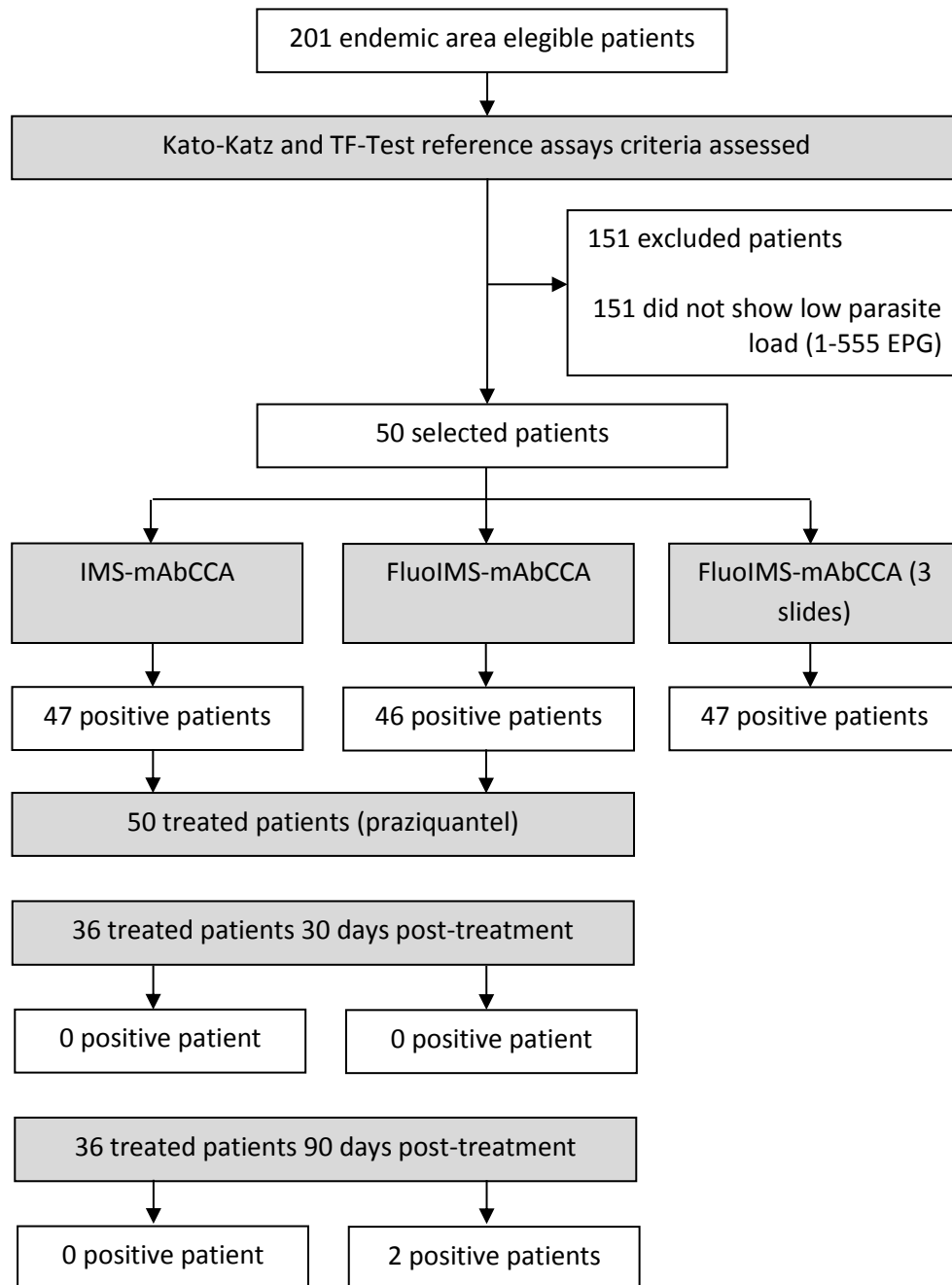

Supplement: Figure S1 — Flowchart. Diagram that represents the sequencing of operations for the prospective study performed in the communities of Buriti Seco and Morro Grande in Pedra Preta, Brazil. (PDF) [file pone.0087777.s001.pdf]
